# Insula Volume and Salience Network Are Associated with Memory Decline in Parkinson Disease: Complementary Analyses of Voxel-Based Morphometry versus Volume of Interest

**DOI:** 10.1155/2016/2939528

**Published:** 2016-02-21

**Authors:** Yan-Ting Lu, Wen-Neng Chang, Chiung-Chih Chang, Cheng-Hsien Lu, Nai-Ching Chen, Chi-Wei Huang, Wei-Che Lin, Ya-Ting Chang

**Affiliations:** ^1^Department of Neurology, Chang Gung University College of Medicine, Kaohsiung 83301, Taiwan; ^2^Department of Biological Science, National Sun Yat-Sen University, Kaohsiung 80424, Taiwan; ^3^Department of Radiology, Chang Gung University College of Medicine, Kaohsiung 83301, Taiwan

## Abstract

*Objective.* We investigated structural brain change in subjects with a clinical diagnosis of Parkinson disease with mild cognitive impairment (PD-MCI) and examined its relationship with memory impairment.* Methods.* Twenty-three PD-MCI patients were enrolled and underwent cognitive evaluation and 3-dimensional T1-weighted imaging. Voxel-based morphometry (VBM) was used to assess brain-behavior correlations and examine the relationship between insula and memory score. VOI methods replicated results obtained from VBM.* Results.* VBM uncovered the notion that memory scores were positively correlated with the gray matter (GM) density in the insular cortex and a significant positive correlation between overall cognitive performance and concentration of GM within the lateral temporal cortex. In VOI analyses, our results suggested a positive correlation between the insula and composite free-recall verbal memory (*ρ* = 0.617, *P* = 0.003) and the delayed free-recall verbal memory subdomain (*ρ* = 0.725, *P* < 0.001). Furthermore, we found a positive correlation between the insula and caudate (*σ* = 0.570, *P* = 0.006) and putamen volume (*σ* = 0.683, *P* < 0.001).* Conclusions.* In patients with PD-MCI, atrophic changes in the insula may be related to memory deficits, and the brain-behavior correlation may be associated with atrophic change in the striatum within the salience network.

## 1. Introduction

Although Parkinson disease (PD) is considered to be a motor disorder, cognitive dysfunctions are known to range from mild cognitive impairment (MCI) to PD dementia [1]. Among nondemented PD patients, MCI in PD (PD-MCI) is common and is associated with an increased risk of developing dementia, which can occur in as many as 80% of PD patients, especially those at an advanced stage [2]. Multiple pathologic processes have been linked to the development of behavioral symptoms in PD patients before the demented stage, including degeneration of basal forebrain cholinergic nuclei and degeneration of the dopaminergic mesocorticolimbic circuits [3], which is thought to induce memory dysfunction [4].

Disruption of dopaminergic modulation in salience network connectivity between the insular cortex and the hippocampus is implicated in memory deficits observed in PD [5]. While long-term memory abnormality in PD results from prefrontal atrophy-induced working memory disorganization [6], a disconnected network between the insula and medial temporal and dopaminergic neurons in the striatum could contribute to impairment of memory consolidation and retrieval in PD [5, 7]. In addition, insula atrophy is associated with memory deficits progression in MCI subjects [8] and cognitive decline, behavioral abnormalities, and sensorimotor disturbances through its reciprocal connections with the medial temporal, striatum, and supplementary motor cortices [9].

In addition to motor function deterioration, PD progression is manifested clinically with cognitive decline and structurally with brain atrophy. Reduced gray matter (GM) volume in PD includes not only the subcortical area (caudate and putamen) but also the cortex (prefrontal and temporal) [10]. By permitting brain-behavior correlations, advances in neuroimaging have provided insight into abnormal cognition in PD and have revolutionized clinical neuropsychology. There have been reports of correlations between verbal memory and ventricular enlargement and between memory impairment and hippocampal atrophy before the demented stage in PD. In another study of demented PD patients, several cortical areas, including the prefrontal gyrus, medial temporal gyrus, and insula, were found to correlate with Raven Colored Progressive Matrices representing the ability of complicated thinking and information storage.

The methods used to assess the relationship between structural change and cognitive impairment in PD include volume of interest (VOI) and voxel-based morphometry (VBM). The VOI method measures the correlation between cognition and manually delineated and anatomically defined regions within the brain [11]. VBM is a fully automated whole brain measurement technique that maps the statistical association between cognition and regional tissue volume or density [12]. Although VBM provides a nonbiased measure of regions within the whole brain measurement, transforming the shape of the brain image during the normalization stage within the VBM analyses may distort the abnormal tissue and artificially inflate the cortical surface. While VBM analyses are less sensitive to shape differences among subjects, the VOI approach provides statistical evaluation of morphometric data of brain cortical regions and has the strength of anatomical validity. However, studies using the VOI method require predefined anatomical regions based on an a priori hypothesis. Thus, it is important to replicate the result shown in the within-voxel concentrations of GM revealed in VBM and the absolute volumes as calculated in VOI analyses.

The aim of the study was to identify the GM atrophic changes that are associated with global cognitive measures and memory performance in PD. We used both VOI and VBM to correlate regional cortical volume and GM density to performance on cognitive scores in PD-MCI patients.

## 2. Methods

### 2.1. Inclusion and Exclusion Criteria

This was a single-center, prospective, observational study. The patients were recruited from the Department of Neurology of Chang Gung Memorial Hospital from the year 2013 to 2014. Twenty-three patients with a clinical diagnosis of idiopathic PD [13] underwent comprehensive neurological and neuropsychological assessments with consensus rendered at a multidisciplinary conference. In cognitive assessment, all of the patients were assessed in the “on” state and were devoid of anticholinergic medication. The inclusion criteria were gradual decline in cognitive ability reported by the patient, cognitive deficits on a global cognitive test, and cognitive deficits that did not interfere significantly with functional independence in daily living and occupational activities [14]. Patients were included only if they had at least two scorings below the cutoff value of the cognitive tests (Mini-Mental State Examination (MMSE) [15], cognitive abilities screen instrument (CASI) and its subdomain [16], and PD dementia- (PDD-) short screen and its subdomain [17]). The exclusion criteria were a diagnosis of PD dementia (PDD) based on the 294.1 criteria for PDD in the Diagnostic and Statistical Manual of Mental Disorders, Fourth Edition, Text Revision (DSM-IV-TR) [18]. The hospital's Human Ethics Committee approved the study protocol, and all of the participants and their authorized caregivers provided written informed consent.

### 2.2. MRI Acquisition and Analysis

MRI was performed using a GE 3T Signa Excite scanner. Structural images were acquired for anatomic reference and clinical diagnosis verification using the following protocols: (1) T2-weighted, turbo spin-echo sequence with repetition time/echo time/number of averages of 4200 ms/101.2 ms/2, 240 × 240 mm field of view, 320 × 224 matrix, and 5 mm axial slice thickness, and (2) T1-weighted, inversion-recovery-prepared, 3-dimensional (3D), spoiled, gradient-recalled acquisition in a steady-state sequence with repetition time/inversion time of 8,600 ms/450 ms, 240 × 240 mm field of view, and 1 mm slice thickness.

#### 2.2.1. VBM Analysis

Using the latest version of SPM8 (Wellcome Department of Imaging Neuroscience, London, United Kingdom), MRIs were segmented into gray matter, white matter, and cerebrospinal fluid images by a unified tissue-segmentation procedure after image-intensity nonuniformity correction. These segmented gray and white matter images were then spatially normalized to the customized template in the standardized anatomic space using DARTEL (Wellcome Department of Imaging Neuroscience) [19]. The gray and white matter volumes within each voxel were preserved by modulating the images using the Jacobean determinants derived from the spatial normalization by DARTEL, and then they were smoothed using a 12 mm FWHM Gaussian kernel. For the correlation analyses with global cognitive performance and memory scores, we used multiple regression analysis and an uncorrected threshold of *P* < 0.001 with a cluster size of >50.

#### 2.2.2. Volumetric Analysis

Using the Individual Brain Atlases Using Statistical Parametric Mapping (IBASPM) (http://www.fil.ion.ucl.ac.uk/spm/ext/) [20], individual 3D T1-weighted MRI images were segmented into different anatomic structures using the Automated Anatomic Labeling atlas [21]. Volumes of interest (VOIs) were defined and expressed in cubic millimeters. Selection of VOI was based on the findings from multiple regression analysis in VBM. The raw regional volume and total intracranial volume (TIV) were calculated.

### 2.3. Neuropsychological Assessment

A trained neuropsychologist administered the tests. Cognitive function was assessed using the Mini-Mental State Examination (MMSE) [22], cognitive abilities screen instrument (CASI) [16], and PDD-short screen [17]. Delayed free-recall verbal memory was used to represent memory encoding and retrieval. We further calculated the composite free-recall verbal memory to recheck the results. This was composed of the immediate free-recall verbal memory score of the PDD-short screen and short-term memory score of CASI.

### 2.4. Statistical Analysis

All values were expressed as mean ± standard deviation (SD). In addition to VBM, the cognitive performance and volumetric data obtained from VOI analyses were assessed using multivariate linear regression analysis. For structural-structural relationships, in addition to Spearman's analysis for VOI analysis, multivariate linear regression analysis was used to test the independent associations between the insula and basal ganglia. To assess the appropriateness of using parametric statistics for these analyses, we used the Kolmogorov-Simonov test to examine the normality, and *P* values >0.05 indicated no significant deviations from normality. All statistical analyses were conducted using the Statistical Package for Social Sciences software package (version 18 for Windows®, SPSS Inc., Chicago, IL) substructures.

## 3. Result

### 3.1. Demographic and Clinical Characteristics

Twenty-three patients completed the study (Table 1). The TIV was 1389.1 ± 132.2 (range: 1197.8–1644.4), and regional volume in descending order was follows: superior temporal gyrus: 12.8 ± 1.8 (range: 10.0–18.7), insula: 11.4 ± 4.7 (range: 8.9–15.7), putamen: 5.9 ± 1.0 (range: 3.5–7.8), caudate: 4.6 ± 0.8 (range: 3.0–6.2), and pallidum: 0.7 ± 0.3 (range: 0.3–1.7).

### 3.2. Memory Scores with Cortical Atrophy Using VBM Analysis

**Figure 1:**
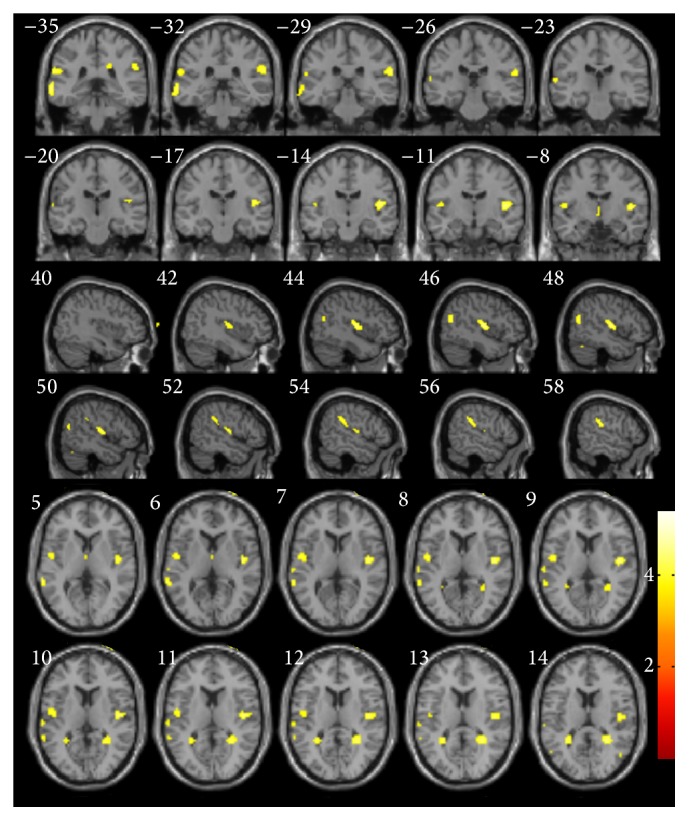
Voxel-based morphometry results showing regions positively correlated with the delayed free-recall verbal memory subdomain of Parkinson disease dementia-short screen (yellow, uncorrected *P* < 0.001).

**Figure 2:**
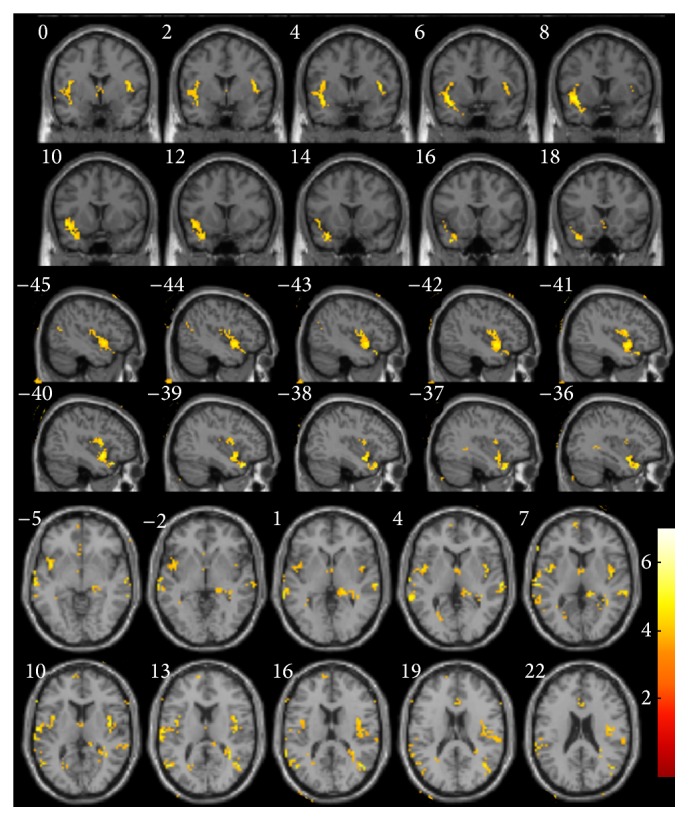
Voxel-based morphometry results showing regions positively correlated with composite free-recall verbal memory (yellow, uncorrected *P* < 0.001).

VBM measures that explain the delayed free-recall verbal memory subdomain of PDD-short screen (Figure 1) were seen in bilateral lateral temporal gyrus and bilateral insula (BA 13). Further rechecking of the result revealed significant positive correlations between composite free-recall verbal memory (Figure 2) and bilateral superior temporal gyrus (BA21/22) and bilateral insula (BA 13) (Table 2).

### 3.3. Memory Scores with Regional Cortical Volume

**Figure 3:**
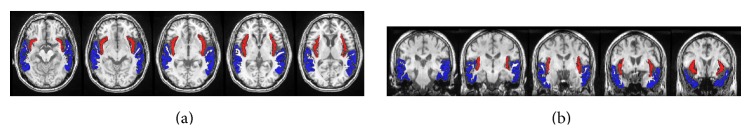
Map of volumes of interest on brain magnetic resonance imaging. (a) Axial view. (b) Coronal view.

Based on the result of VBM study, we chose insula and superior temporal gyrus as our VOI (Figure 3). In VOI analysis, there was a significant relationship between TIV-adjusted insula volume and memory scores. After controlling for age and education in linear regression analysis, larger insula volume was associated with better performance in the composite free-recall verbal memory (*β* coefficient = 0.832, *P* = 0.003) and delayed free-recall verbal memory subdomains (*β* coefficient = 0.809, *P* < 0.001). None of these memory score volumes were associated with regional volume in superior temporal gyrus (*P* > 0.05).

### 3.4. Cognitive Performance with Cortical Atrophy Using VBM and VOI Analysis

In VBM analysis, MMSE, CASI, and PDD-short screen did not associate with gray matter integrity in any cortical region. In the VOI analyses, we observed significant positive correlations between cognitive performance and TIV-adjusted insula volume. After controlling for age and education, a larger insula volume was associated with better performance in CASI (*ρ* = 0.498, *P* = 0.022), MMSE (*ρ* = 0.463, *P* = 0.034), and PDD-short screen (*ρ* = 0.463, *P* = 0.034). None of the other regional cortical volumes were associated with cognitive performance (*P* > 0.05).

### 3.5. Examining the Relationship between Insular Volume and Basal Ganglion Volume

**Figure 4:**
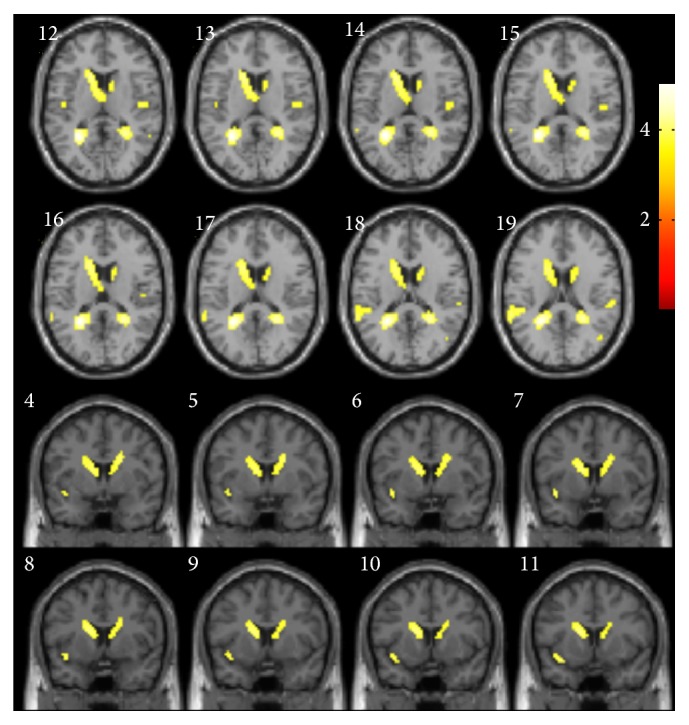
Voxel-based morphometry results showing regions positively correlated with normalized ratio of insula volume (yellow, uncorrected *P* < 0.001).

As the dopaminergic neuron in the striatum was thought to modulate insula activity within the salience network, the relationship between insular volume and the striatum was examined. After controlling for age, data for the Spearman correlations between insulae with basal ganglia substructure volumes (Table 3) revealed a significant correlation between TIV-adjusted insula volume and volume of the TIV-adjusted caudate (*σ* = 0.515, *P* = 0.014) and TIV-adjusted putamen (*σ* = 0.522, *P* = 0.013). The 3D statistical maps showed TIV-adjusted insular volume correlated strongly with gray matter integrity in the bilateral caudate nuclei (Table 3, Figure 4).

## 4. Discussion

This study investigated the associations between cognitive performance and GM density in PD-MCI patients using VBM and related VOI volumetric measurements, from which there were several major findings. First, we established that memory impairment in PD is associated with loss of insular volume, using VBM as well as VOI-based methods. There were (1) a positive correlation between memory performance scores and insular volume and (2) an association between insular volume and striatum volume among the PD patients in the present study. While both VOI and VBM analyses showed a positive correlation between the insula and caudate, our results showed an association between the 2 interconnected structures within the salience network. Second, lateral temporal volume was positively associated with global cognitive performance, and the results obtained in further VOI analyses replicated those from VBM studies. Third, the insula volume was insufficient to explain the global cognitive deficits, while relationships were seen only in VOI volumetric correlations. Using VBM, only a small cluster of the right insulae was found to be associated with PDD-short screen.

### 4.1. Role of Insula Volume in Memory Scores

The association of reduced insula volume with memory deficits and the positive correlation with caudate volume in PD-MCI patients suggests that volume changes of structure within the salience network contributed to memory dysfunction. There has been a growing interest in exploring the crucial role of the insula in PD due to the reciprocal projections to functional regions of the striatum [9]. Striatal dopamine depletion in PD was reported to be associated with loss of dopaminergic modulation in the insula of PD-MCI patients [5]. The striatum is functionally and structurally connected to the insula and has been described as a cognitive hub and key region of a salience network [5]. A correlation between the D2 receptor within the insula and memory scores has been shown in PD-MCI patients [5]. The salience network is crucial for its interaction with the medial temporal lobe and is engaged in memory encoding and retrieval in PD patients [5, 7], through switching between the central executive and default mode network [23]. Pathways from the striatal projection to the insula are difficult to represent in volumetric analyses of positive correlations of the insula with the caudate. While the insula is an important target underlying executive function and memory, disruption of the normal function of the insula as well as the salience network occurs in early disease stages [24, 25] and has an impact on cognition in PD-MCI. As our results showed that the insula was positively correlated with caudate volume and positively related to memory performance, the brain-behavior correlations provided in both VBM and VOI analysis pointed to the importance of the insula.

### 4.2. Insula Volume Contribution to Global Cognitive Decline in PD-MCI

The insula is highly interconnected with the basal ganglia and many cortical regions including the frontal, temporal, and parietal regions [26]. Functional roles of the insula may include cognitive/affective and somatosensory awareness through the corticocortical circuitry [27]. Thus, while the insula is able to interact with multiple brain networks, the corticostriatal circuitry has been the main anatomical target of many of the symptoms of PD [9]. The insula has abnormal activation patterns in PD patients during cognitive tasks [28], and the cortical regions, including the prefrontal cortex and anterior cingulate cortex, which functionally connect with the insula also show abnormal activation in patients with cognitive dysfunction [28]. As atrophy in angular and middle occipital gyri, middle frontal and precentral gyri, supplementary motor and inferior frontal gyri, instead of insula cortex, has been reported in PD-MCI patients [29], we suggested insula volume could affect cognition in PD-MCI even before appearance of gray matter atrophy in insula. However, differences in the atrophic pattern may vary with races, years of PD evolution, severity of motor disability, and educational level of PD [29, 30]. Using photon emission tomography imaging, loss of dopamine receptor availability in the right insula was seen to contribute to cognitive deficits in PD-MCI [31]. Furthermore, the dopaminergic pathway in bilateral insula of PD-MCI patients was reduced compared with that of healthy controls. The small cluster of the right insulae that was found to be associated with PDD-short screen using VBM methods was insufficient to explain the global cognitive deficits. The relationship between the insula and overall cognitive testing of CASI and MMSE in VOI volumetric correlations in the present study suggests that when taking bilateral insula into consideration, insular dysfunctions are associated with cognitive deficits in PD-MCI patients.

### 4.3. Lateral Temporal Volume Associated with Cognitive Scores

Significant cortical thinning in the middle temporal gyrus was seen in PD patients without dementia [32]. MMSE, representing overall cognitive performance, showed a positive correlation with thickness of the temporal cortices in another study [33]. In addition to volumetric data, reduced cerebral blood flow in the lateral temporal regions was found in PD patients [34]. Our findings with respect to the association between overall cognitive performance and the lateral temporal volume are in concordance with a recent cross-sectional study of PD patients with a different cognitive disease stage [33] and another study with nondemented PD patients [32].

VBM provides a brain-behavior measurement after transforming the shape of the brain image, which may distort the abnormal tissue, and volumetric analysis using the VOI technique after the GM segmentation-based volume quantification method replicates the results obtained from the VBM method. Although our GM segmented VOI technique required manual rechecking and thus was labor-intensive to ensure correct coregistration in each subject, the VOI approach provides landmark-based analyses and has the strength of anatomical validity. Both VBM and VOI analyses showed a direct association of the lateral temporal volume with CASI and MMSE in this study, which reinforces the significance of the crucial role of the lateral temporal volume in PD-MCI.

## 5. Limitations and Conclusion

Several limitations in our study need to be further discussed. First, we used memory subdomain scores of CASI and PDD-short screen to assess memory function. Different memory processes, such as encoding, consolidation, and retrieval, were not dissociated and discussed separately. The relationship between memory performance and insular cortex was reported after careful statistical examination by VBM and VOI methods and rechecking 3 separate memory subdomain scores of CASI and PDD-short screen. In addition, there might have been selection bias in which only PD-MCI patients were enrolled in the study, and among PD-MCI patients, we did not select patients with exclusively impaired memory performance in PD-MCI. Furthermore, PD-MCI patients in this study only fulfilled the guidelines for level I categories. However, patient selection involved the avoidance of overall volume atrophy, which would obscure exploration of the association between regional cortical atrophy and cognitive performance.

In conclusion, our study indicates that insular volume atrophy, which was distantly interconnected with the striatum, may contribute to memory deficits in PD-MCI patients. While memory performance is related to insular volume, changes in lateral temporal volume could contribute to overall cognitive decline in PD-MCI patients.

## Figures and Tables

**Table 1:** Clinical and demographic characteristics of the PD-MCI patients.

	PD-MCI, *n* = 23, mean (SD)
Age (yr)	68.1 ± 8.4
Education (yr)	6.5 ± 1.2
Men (%)	47.8%
PD duration (yr)	4.8 ± 2.2
Hoehn and Yahr	Stage 1 = 7
	Stage 2 = 15
	Stage 3 = 1
UPDRS-III	29.8 ± 16.6
MMSE	24.0 ± 3.8
CASI	77.9 ± 11.6
Concentration	5.8 ± 2.9
Attention	7.0 ± 1.0
Orientation	16.4 ± 2.3
Long-term memory	9.8 ± 0.6
Short-term memory	8.0 ± 3.3
Abstraction	7.7 ± 2.6
Visual construction	7.7 ± 2.6
List-generating fluency	6.3 ± 2.1
Language abilities	8.8 ± 1.5
PDD-short screen	12.9 ± 4.6
Immediate free-recall verbal memory	3.7 ± 1.4
Alternating verbal fluency	1.4 ± 0.8
Questionnaire	3.9 ± 1.1
Clock-drawing task	2.2 ± 1.6
Delayed free-recall verbal memory	2.0 ± 1.7

CASI: cognitive abilities screening instrument; MMSE: Mini-Mental State Examination; PD: Parkinson disease; PDD: PD dementia.

**Table 2:** Location, AAL coordinates, and statistical significance of the regions showing the associations of cognitive performance and gray matter atrophy.

Associations of cognitive scores and gray matter atrophy	Region	BA	Coordinates (mm)			*P*	*T* value	*F* value
			*X*	*Y*	*Z*			
MMSE	Left superior temporal gyrus		−49.37	18.58	−20.45	<0.001		24.47
	Left middle temporal gyrus	Left BA 21	−65.38	−14.74	−9.35	<0.001		24.15
	Left superior temporal gyrus	Left BA 42	−65.35	−24.27	9.39	<0.001		23.82
	Right inferior temporal gyrus		55.56	−60.45	−23.87	<0.001		19.10
	Right middle temporal gyrus		67.59	−28.61	−17.99	<0.001		17.44

CASI	Left superior temporal gyrus	Left BA 38	−43.38	21.68	−24.37	<0.001	5.11	
	Left middle temporal gyrus		−7.40	−27.59	−14.71	<0.001	4.76	
	Left superior temporal gyrus	Left BA 42	−65.35	−24.32	11.39	<0.001	4.53	
	Right superior temporal gyrus	Right BA 22	67.63	−23.15	1.17	<0.001	4.01	
	Right middle temporal gyrus		68.60	−28.67	−15.99	<0.001	3.66	

PDD-short screen	Left middle temporal gyrus	Left BA 39	−54.37	−61.49	16.32	0.001	3.62	
	Right superior temporal gyrus		66.64	−24.20	3.15	0.001	3.80	
	Right insula		45.66	−10.33	8.58	0.001	3.77	

Composite free-recall verbal memory	Right middle temporal gyrus	Right BA 21	67.60	−33.72	−14.13	0.001	6.32	
	Right superior temporal gyrus		66.64	−25.26	5.12	0.001	5.32	
	Right insula gyrus	Right BA 13	42.67	0.67	8.8	0.001	4.95	
	Left temporal pole	Left BA 38	−39.38	22.76	−27.35	0.001	5.74	
	Left superior temporal gyrus		−62.34	−15.30	10.64	0.001	5.62	
	Left middle temporal gyrus	Left BA 19	−52.38	−63.38	12.26	0.001	5.12	
	Left middle temporal gyrus		−52.36	−59.57	19.37	0.001	3.96	
	Left insula gyrus	Left BA 13	−43.31	3.31	−2.04	0.00	5.88	

Delayed free-recall verbal memory	Right insula	Right BA 13	44.60	−9.60	3.13	0.001	3.94	
	Left insula	Left BA 13	−48.48	−12.39	9.6	0.001	3.77	
	Right insula	Right BA 13	40.66	−13.38	10.50	0.001	3.62	
	Right insula		93.73	−12.34	−67.97	0.001		
	Right insula	Right BA 12	48.48	−12.34	8.3	0.001	4.46	
	Left inferior frontal gyrus		−34.38	11.51	−18.67	0.001	4.08	
	Right supramarginal gyrus		60.67	−28.70	21.04	0.001	4.27	
	Left middle temporal gyrus		−65.38	−37.04	1.03	0.001	4.46	
	Left superior temporal gyrus		−61.34	−34.63	22.09	0.001	4.17	
	Right middle temporal gyrus		46.63	−68.68	19.94	0.001	4.15	
	Left rolandic opercular gyrus		−50.34	−6.23	7.87	0.001	4.03	

BA: Brodmann area; CASI: cognitive abilities screening instrument; MMSE: Mini-Mental State Examination; PDD: Parkinson disease dementia.

**Table 3:** Relationship between TIV-adjusted insular volume and basal ganglia substructures.

Region	Age-adjusted partial correlation (*σ*)	BA	Coordinates (mm)			*T* value	*P*
Caudate	0.515^*∗*^	Left caudate	−14.31	16.59	13.45	4.34	0.001
		Right caudate	11.68	7.57	13.15	3.82	0.001
Putamen	0.522^*∗*^						
Pallidum	0.304						

BA: Brodmann area; TIV: total intracranial volume.
